# Gene Transfer to Chicks Using Lentiviral Vectors Administered via the Embryonic Chorioallantoic Membrane

**DOI:** 10.1371/journal.pone.0036531

**Published:** 2012-05-11

**Authors:** Gideon Hen, Sara Yosefi, Dmitry Shinder, Adi Or, Sivan Mygdal, Reba Condiotti, Eithan Galun, Amir Bor, Dalit Sela-Donenfeld, Miriam Friedman-Einat

**Affiliations:** 1 Ministry of Agriculture, Volcani Center, Bet-Dagan, Israel; 2 Koret School of Veterinary Medicine, The Robert H. Smith Faculty of Agriculture, Food & Environment, The Hebrew University of Jerusalem, Rehovot, Israel; 3 Goldyne Savad Institute of Gene Therapy, Hadassah-Hebrew University Medical Center, Jerusalem, Israel; University of Connecticut, United States of America

## Abstract

The lack of affordable techniques for gene transfer in birds has inhibited the advancement of molecular studies in avian species. Here we demonstrate a new approach for introducing genes into chicken somatic tissues by administration of a lentiviral vector, derived from the feline immunodeficiency virus (FIV), into the chorioallantoic membrane (CAM) of chick embryos on embryonic day 11. The FIV-derived vectors carried yellow fluorescent protein (YFP) or recombinant alpha-melanocyte-stimulating hormone (α-MSH) genes, driven by the cytomegalovirus (CMV) promoter. Transgene expression, detected in chicks 2 days after hatch by quantitative real-time PCR, was mostly observed in the liver and spleen. Lower expression levels were also detected in the brain, kidney, heart and breast muscle. Immunofluorescence and flow cytometry analyses confirmed transgene expression in chick tissues at the protein level, demonstrating a transduction efficiency of ∼0.46% of liver cells. Integration of the viral vector into the chicken genome was demonstrated using genomic repetitive (CR1)-PCR amplification. Viability and stability of the transduced cells was confirmed using terminal deoxynucleotidyl transferase (dUTP) nick end labeling (TUNEL) assay, immunostaining with anti-proliferating cell nuclear antigen (anti-PCNA), and detection of transgene expression 51 days post transduction. Our approach led to only 9% drop in hatching efficiency compared to non-injected embryos, and all of the hatched chicks expressed the transgenes. We suggest that the transduction efficiency of FIV vectors combined with the accessibility of the CAM vasculature as a delivery route comprise a new powerful and practical approach for gene delivery into somatic tissues of chickens. Most relevant is the efficient transduction of the liver, which specializes in the production and secretion of proteins, thereby providing an optimal target for prolonged study of secreted hormones and peptides.

## Introduction

For several decades now, great effort has been invested in producing transgenic chickens [1]–[3]. Inherited biological and anatomical obstacles to avian transgenesis, arising from the unique anatomy of the avian reproductive system and a low rate of genomic incorporation of foreign DNA, have prevented the adaptation of protocols routinely used in mice. Therefore, alternative approaches were developed for chicken transgenesis, such as: (i) infection of primordial germ cells by viral injection into the subgerminal cavity of the newly laid egg [3]–[5], or at a later stage of development, upon primordial germ cell migration to the gonads through the circulation on embryonic day 2.5 (E2.5) [6]; (ii) injection of *in vitro*-modified embryonic stem cells or primordial germ cells, either employing non-viral vector systems, which allow insertion of large DNA fragments [7]–[10], or by utilizing viral vectors [11]. However, production of transgenic chickens using these approaches is much less efficient and more complex than transgenesis in other model animals, thereby preventing their routine use for research purposes.

In contrast to the high complexity of the existing techniques for stable transgenesis in adult birds, transient transgenesis in chick embryos is widely used for developmental biology studies (for review see [12]). However, these approaches are not compatible with long term development and hatch.

Among the reported viruses used for transduction of chicken cells, lentiviruses appear to be the most efficient [4], [11]. Efficacy of lentiviral vectors in several clinical trials has been recently reported, such as use for gene-therapy studies in human cancer patients [13]. Lentiviral vectors are considered the preferred vector system for gene therapy due to their competence in transducing a wide variety of cell types, their unique ability to integrate into the genome of both dividing and non-dividing cells, and their considerable resistance to gene silencing, resulting in stable and long-term transgene expression [14]–[18].

The chorioallantoic membrane (CAM) is the site of respiratory gas exchange, calcium transport from the eggshell, acid-base homeostasis in the embryo, and ion and H_2_O reabsorption from the allantoic fluid. It consists of fused allantois and chorion membranes and is rich in blood vessels. Its position proximal to the shell membrane renders the CAM vasculature highly attractive for a variety of research purposes, such as the delivery of cells or chemicals to test tumor chemosensitivity [19], to study angiogenesis and metastasis [20], to evaluate drug-delivery systems in preclinical studies [21], and to assess the safety of cosmetic formulations [22].

In the current study, we present a new approach to gene delivery into somatic tissues of chickens *via* administration of lentiviral particles carrying either yellow fluorescent protein (YFP) or recombinant alpha-melanocyte-stimulating hormone (α-MSH) genes, into the CAM on embryonic day 11 (E11). Analysis of post-hatch chicks showed that all of them expressed the transgene in various tissues, with highest levels of expression in the liver and spleen and lower levels in the brain, kidney, heart and breast muscle. The combination of a simple injection into the embryonic CAM and the use of an advanced feline immunodeficiency virus (FIV)-derived vector system comprise a unique and powerful method for gene delivery into somatic tissues of chicks.

## Materials and Methods

### Ethics Statement

All procedures were carried out in accordance with the National Institutes of Health Guidelines on the Care and Use of Animals and approved by the “Animal Experimentation Ethics Committee” of the ARO, Volcani Center (Protocol #356-0479-06).

### Eggs, Incubation and Hatching Conditions

Fertile White Leghorn eggs were purchased from a local husbandry (Wolf-Weisman, Sitriya, Israel). Incubation was performed in a standard egg incubator, at 37.8°C and 56% relative humidity (RH). Eggs were incubated with their narrow end facing down, and rotated 90° once per hour. On E18, eggs were transferred to the hatching compartment in the incubator and incubation was continued at 37.8°C and 70% RH. Hatchability was 90% for untreated eggs.

### Plasmids

pLionII-YFP (http://www.stanford.edu/group/nolan/retroviral_systems/felix_maps.html), which contains the gene encoding YFP driven by the CMV promoter, was kindly donated by Garry Nolan (Stanford University, Palo Alto, CA).

pLionII- α-MSH was constructed by digesting pLionII (Addgene, plasmid #1730) with EcoR*V* and inserting, downstream of the CMV promoter, a blunted BamH*I* fragment containing the sequence encoding human α-MSH from the plasmid pACTH1-17 (kindly donated by Dr. M.L. Hedley [23]). The α-MSH coding sequences in this construct are composed of selected segments of the human pro-opiomelanocortin (POMC) gene (signal peptide, sorting peptide, partial junction peptide, α-MSH-encoding sequence and a 12 base-pairs (bp) sequence encoding the α-MSH amidation signal [23]). The full sequence of pLionII-α-MSH (pLionII-pACTH1-17) was submitted to GenBank under accession number: BankIt1497321 seq JQ086322.

### Production of Viral Particles

Viral particles were produced as described previously [16]. Briefly, HEK293T cells were co-transfected with 8.4 µg of the transfer vector (pLionII-YFP or pLionII- α-MSH), 14 µg packaging vector pCPRΔEnv (Addgene, plasmid #1732), and 5.6 µg of the envelope vector pCI-VSVG (Addgene, plasmid #1733) per 10 cm diameter plate, using 75 µl of Fugene reagent (Roche Diagnostics, Pharma, Petach-Tikva, Israel). The VSV-G envelope has a broaden viral tropism including avian cells [4]. FIV transducing units (TU) were determined by titration of HEK293T transduction using flow cytometry for FIV-YFP, as well as by real-time quantitative (q) PCR (see below) for FIV-α-MSH, using canonical packaging signal (ψ) primers F: 5′- CGGACTCGAGCTCATAATCAAGT-3′, R: 5′-TGTCCCTCGGCGAATCTC-3′. A known amount of pLionII- α-MSH plasmid was used as a reference for calibration.

### Cell Culture Conditions and Lentiviral Transduction

HEK293T cells were grown in Dulbecco’s modified Eagle’s medium (DMEM) supplemented with 10% (v/v) fetal calf serum (FBS), 2 mM glutamine, 100 µg/ml streptomycin and 100 U/ml penicillin (Biological Industries, Bet-Haemek, Israel), at 37°C in an atmosphere containing 5% CO_2_. For transduction with the lentiviral vectors, 1×10^6^ cells were seeded in a six-well plate (Corning, Sigma-Aldrich, Rehovot, Israel) and transduced 1 day later with FIV-YFP or FIV-α-MSH (2×10^5^ TU/well), estimated as 0.1 TU/cell at the moment of transduction.

### *In vitro* Transduction of Chicken Cells in Primary Cultures with FIV-YFP

Liver, spleen, kidney, brain, heart and breast muscle (pectoralis) were excised from E11 chicken embryos and dissociated with 2 mg/ml collagenase II, 0.15 mg/ml DNase I, 100 µg/ml streptomycin and 100 U/ml penicillin in HBS (20 mM Hepes, 137 mM NaCl, 3 mM KCl, pH 7.4) for 20 min at 37°C, with pipetting every 5 min. Dissociated cells were filtered through a 70 µm cell strainer (BD Falcon, Bactlab Diagnostics Ltd, Caesarea, Israel) to enrich for a single-cell suspension, and treated with red blood cell (RBC) lysing buffer (Sigma-Aldrich). Enrichment against fibroblasts was performed by pre-plating the cells for 20 min in a standard 24-well plate (Corning) before transferring the non-attached cells to new plates coated with 0.01% (w/v) calf skin collagen solution (Sigma-Aldrich). All cell types were cultured in DMEM supplemented with 10% FBS. Liver culture medium was also supplemented with ITS (Sigma-Aldrich), containing 5 µg/ml recombinant human insulin, 5 µg/ml human transferrin and 5 ng/ml sodium selenite. Twenty-four hours after plating, cells were transduced by adding 300 µl of medium containing 2×10^4^ TU of FIV-YFP, supplemented with polybrene (8 µg/ml; Sigma-Aldrich) for 24 h. Two days after transduction, cells were washed with PBS and fixed with 4% (w/v) paraformaldehyde (PFA) for 10 min or analyzed by flow cytometry. Digital images were taken using a Nikon Eclipse TS100 microscope, equipped with an Olympus DP72 camera and Olympus DP controller software.

### Estimation of Transduction Efficiency by Flow Cytometry

To estimate the percentage of YFP-expressing cells in cell culture and chicken tissues, cells were analyzed using the LSRII flow cytometer with FACSDiva software (BD Biosciences, Ness-Ziona, Israel). Cultured cells (>1.2×10^5^) were trypsinized, washed with PBS and immediately analyzed. For the liver tissue, pieces of excised liver were minced with scissors and a single-cell suspension was generated as described above and immediately analyzed.

### Nucleic Acid Extraction

DNA was extracted from cells in culture or tissues using DNA lysis buffer (10 mM Tris, 10 mM EDTA, 0.5% SDS, 200 µg/ml proteinase K) at a ratio of 0.5 ml per well or per 0.05 g tissue, and placed at 55°C in a rotating shaker for 5 h or overnight, respectively. RNA was eliminated by incubation of the DNA samples with RNase at a final concentration of 25 µg/ml at 37°C for 1 h, followed by extraction with phenol-chloroform and ethanol precipitation.

Total RNA was extracted using RNAzol B solution (Tel-Test Inc., Talron, Rehovot, Israel) according to the manufacturer’s instructions. Briefly, 0.5 g of tissue, or subconfluent culture from a six-well plate, was homogenized in 0.5 ml RNAzol B solution using a Polytron PT3000 homogenizer (Kinematika, Labotal, Jerusalem, Israel). After centrifugation, the RNA in the upper phase was re-extracted with a phenol-chloroform solution and precipitated with ethanol.

Turbo DNA-free kit was used for DNA elimination according to the manufacturer’s protocol (Ambion, Agenteck, Tel-Aviv, Israel). The concentration and integrity of the extracted RNA were determined by spectrophotometry and gel electrophoresis, respectively.

### PCR and RT-PCR

PCR was carried out in a 20 µl final volume, using genomic DNA (50 ng) or cDNA (see below), with primers for the transgenic α-MSH (RT-PCR F: 5′-TGGAAGATGCCGAGATCGTGC-3′, RT-PCR R: 5′- TCCTTACCGCTTCTTGCCCAC -3′) or glyceraldehyde 3-phosphate dehydrogenase (GAPDH) (F: 5′-GGAGCCAAAAGGGTCATCATC-3′, R: 5′-AGGTCAGGTCCACCACTGACA-3′), generating amplicons of 234 bp and 397 bp, respectively. The α-MSH forward primer is specific for the junction between the viral and POMC sequences (Figure S1), and this amplification is therefore specific for the recombinant α-MSH. The cycling protocol was: 94°C denaturation for 3 min, followed by five cycles of 94°C denaturation (30 s), 65°C annealing (60 s) and 72°C extension (30 s), another five cycles as above but with annealing temperature of 60°C, and an additional 25 cycles with annealing temperature of 55°C. RT reactions were carried out using 2 µg total RNA as template and a high-capacity cDNA reverse transcription kit (Applied Biosystems, Agentek, Yavne, Israel) according to the manufacturer’s protocol.

### Detection of FIV Integration into the Chicken Genome using Repetitive DNA (CR1) PCR

Genomic DNA was prepared 14 days after *in vitro* transduction of primary cell cultures from E11 embryo liver and muscle tissues, with FIV-YFP at multiplicity of infection (MOI) of one. The initial PCR was performed in a 20 µl reaction volume, using 100 ng template genomic DNA with one of the following CR1 primers: CR1-1 F: 5′-TGGTTGGGTTGGAAGGGACC-3′, R: 5′ GGTCCCTTCCAACCCAACCA-3′; CR1-3 F: 5′-TCCATGGCCTTGGGCACATC-3, R: 5′-GATGTGCCCAAGGCCATGGA-3′, in combination with one of the long terminal repeat (LTR)-specific primers encoded by FIV-YFP: right LTR F: 5′-GGAGTCTCTTTGTTGAGGAC-3′, left LTR R: 5′-CGAAGTTCTCGGCCCGGATTCC-3′. Control reactions contained the same DNA templates and LTR-specific primers but lacked the CR1 primers. Altogether, for each template DNA, eight CR1-LTR reactions for the first PCR and two control LTR reactions were performed. Additional controls consisted of DNA template from non-transduced liver and muscle cells. The protocol of amplification was as described above but elongation was extended to 8 min at 68°C to allow for long range amplification, using the BIO-X-ACT Long DNA Polymerase (Bioline, Origolab, Jerusalem, Israel). A second, “nested” PCR was carried out with 1 µl of a 1∶1000 dilution of the first PCR product as template and nested primers for the left and right FIV-LTRs: left nested LTR F: 5′-GGAGTCTCTTTGTTGAGGAC-3′, R: 5′-ATTCCGAGACCTCACAGGTA-3′ and right nested LTR F: 5′-CTCCCTTGAGGCTCCCACAG-3′, R: 5′- CGAAGTTCTCGGCCCGGATTC**-**3′.

### qPCR

qPCR was performed using Fast SYBR Green Master Mix (Applied Biosystems) according to the manufacturer’s protocol. The cDNA templates of the indicated tissues (2 µl) were used with primers specific for the recombinant sequence of α-MSH (F: 5′-TTGCTGGCCTTGCTGCTT-3′ and R: 5′-GCACTCCAGCAGGTTGCTTT-3′), resulting in a 101 bp fragment. The forward primer was designed according to the adjacent virus-derived sequence, placed upstream of the ATG signal. Therefore, these primers were specific to the exogenous α-MSH sequence (the position of the primers is illustrated in Figure S1). The YFP primers used were F: 5′-TCAGCTCGATGCGGTTCAC-3′ and R: 5′-GTCCAGGAGCGCACCATCT-3′, giving rise to a 99-bp amplicon. Gene expression was normalized to a housekeeping gene encoding chicken ribosomal 17 S protein (accession number X07257). The specific primers F: 5′- GACCCGGACACCAAGGAAAT-3′ and R: 5′- GCGGCGTTTTGAAGTTCATC-3′ give rise to a 100 bp product. Standard curve slope for these primers was -3.331 and R^2^ was 0.994. Another set of primers for the 17 S ribosomal protein gene (F: 5′-AAGCTGCAGGAGGAGGAGAGG-3′ and R: 5′- GGTTGGACAGGCTGCCGAAGT-3′, giving a 136-bp amplicon, gave similar results (not shown). Normalization with GAPDH (F: 5′-GCACCACCAACTGCCTGG-3′ and R: 5′-CTGTGTGGCTGTGATGGCAT-3′ giving a 100 bp amplicon) gave essentially similar results, but expression of GAPDH was lower in the spleen than in the liver (not shown). PCRs were performed using the StepOnePlus™ Real-Time PCR System (Applied Biosystems) with the following cycling protocol: 95°C denaturation for 10 min, followed by 40 cycles of 95°C denaturation (15°s), 60°C annealing (40°s) and 72°C extension (30 s). At the end of the real-time PCR, a melting curve was determined to verify the presence of a single amplicon. Relative quantification was calculated using the 2^−ΔCt^ method. All experiments were run in triplicates and repeated until variations between repeats were below 10%. The PCR products were purified and fragment identity was determined by sequencing.

### Immunofluorescence

Tissue samples were fixed in 4% PFA in PBS overnight at 4°C. For analysis of paraffin sections, tissues were embedded in paraffin and sliced into 10-µm sections using a Leica RM2255 microtome. Slides were treated with 3% H_2_O_2_ in PBS for 30 min at room temperature, blocked with 10% goat serum in PBS for 1 h, and incubated with rabbit anti-GFP (diluted 1∶500, Invitrogen, Rhenium, Israel) at 4°C overnight. Following washes in PBS-0.05% Tween (PBS-T), slides were incubated with goat anti-rabbit Alexa 488 antibody (diluted 1∶500, Invitrogen) at room temperature for 1 h, and washed with PBS-T. For labeling of smooth muscle actin (SMA), slides were similarly incubated with mouse anti-SMA antibody (diluted 1∶200, DAKO, Enco, Petach-Tikvah, Israel), followed by goat anti-mouse Alexa 594 antibody (diluted 1∶500, Invitrogen). Finally, sections were stained with 4′,6-diamidino-2-phenylindole (DAPI) and digital images were taken using a Nikon Eclipse e400 upright microscope, equipped with an Olympus DP72 camera and Olympus DP Controller software. Confocal images were obtained using an Olympus IX81 inverted laser scanning microscope with Fluoview-500 software.

For whole-mount immunofluorescence analysis of excised tissue pieces (sized 5–10 mm), double-labeling was performed according to Alanentalo *et al.* [24], except that rabbit anti-GFP (diluted 1∶700, Invitrogen) and mouse anti-SMA (diluted 1∶200) antibodies were used, followed by goat anti-rabbit Alexa 488 or anti-mouse Alexa 594 antibodies (both diluted 1∶700). Clearing of the double-labeled pieces of liver tissue with benzyl alcohol and benzyl benzoate (Sigma-Aldrich) was performed as described previously [24]. Images were taken using the Olympus SZX16 epi-fluorescent stereomicroscope equipped with an Olympus DP72 camera.

### Analysis of Cell Viability and Proliferation

Apoptosis of YFP-expressing cells was examined in paraffin sections using the In Situ Cell Death Detection Kit, TMR red (Roche Diagnostics; Dyn Diagnostics Ltd., Caesarea, Israel), which stains nicked DNA by end labeling of terminal deoxynucleotidyl transferase dUTP (TUNEL). Cell proliferation was demonstrated by immunofluorescence using anti-proliferating cell nuclear antigen (anti-PCNA, diluted 1∶200, Dako) followed by staining with goat anti-mouse Alexa 594 antibody (diluted 1∶500).

### Detection of α-MSH Peptide by Radioimmunoassay (RIA)

Production of α-MSH peptide by HEK293T cells was examined 10 days after transduction with FIV-α-MSH vectors, using a RIA kit (EURIA- α-MSH; Euro Diagnostica AB, Malmo, Sweden) according to the manufacturer’s protocol. HEK293T cells transduced with FIV-YFP vectors served as a control. Conditioned medium of 10 cm culture dish, incubated for 24 h with 6 ml of Opti-MEM culture medium (Gibco, Rhenium), was collected and cell extracts were prepared using 300 µl of CHAPS lysis buffer (1% w/v CHAPS, 30 mM Tris pH 7.5, 150 mM NaCl, Complete protease inhibitor cocktail from Roche Diagnostics, 50 µg/ml DNase and 10 µg/ml RNase. After 10 min incubation on ice, cell debris was pelleted by centrifugation at 10,000 *g* for 10 min. Supernatant was kept at −20°C until assay. Cell extracts and conditioned media were analyzed in 100 µl samples diluted 3- and 10-fold (using the supplied dilution buffer) and results were calibrated with the α-MSH titration curve supplied with the RIA kit.

### Statistical Analysis

Statistical analyses were performed by one-way ANOVA and Tukey-Kramer honestly significant difference test. Comparison of hatching rates of the different experimental treatments was performed using the chi-squared test.

## Results

### Transgene Expression in Transduced Cells of Embryonic Primary Cultures

**Figure 1:**
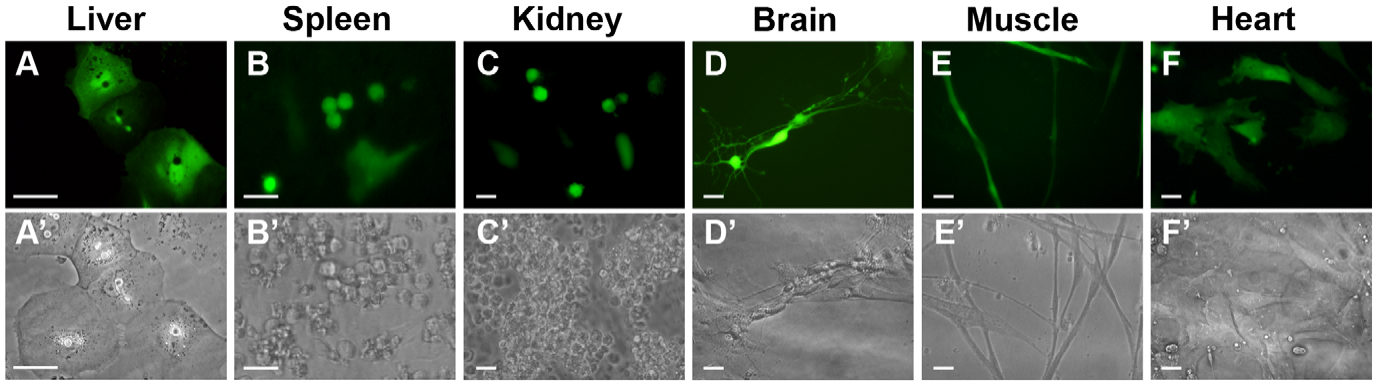
YFP expression following transduction of primary cultures of chick embryonic cells with FIV-YFP vectors. Primary cell cultures from E11 chick embryos were transduced with FIV-YFP (2×10^4^ TU/well, in a 24-well plate) and YFP-expressing cells (green) were detected by fluorescence microscopy, 48 h after transduction (**A–F**). The corresponding bright-field micrographs of the same cultures (**A**′**–F**′) demonstrate the tissue-specific morphology of the cells. Scale bar = 20 µm. Rate of transduction in each culture, determined by FACS analysis, is shown in Figure S2.

To test the efficacy of FIV particles for gene delivery into somatic cells of chick embryos, primary cultures of liver, spleen, kidney, brain, heart and muscle tissues were prepared from E11 chick embryos, and transduced with FIV-YFP (6.5×10^4^ TU/ml). Expression of YFP was demonstrated 48 h later in all transduced cell types (Figure 1). Quantification of the results by flow cytometry (FACS) analysis revealed 1.3, 1.2, 2.8, 2.7, 2 and 4.7% YFP-expressing cells in the liver, spleen, kidney, brain, muscle and heart primary cultures, respectively (Figure S2). These results provide a first demonstration of FIV particle transduction of various types of primary cultured cells derived from chick embryos *in vitro*.

### *In vivo* Transduction of Chicken Embryonic Tissues with Lentiviral Particles

**Figure 2:**
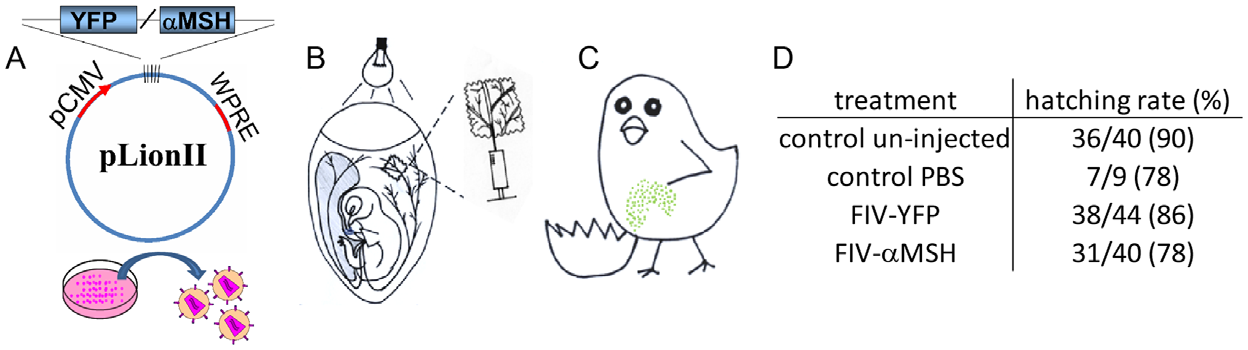
Schematic representation of the gene-transfer procedure used to introduce foreign genes into chicken tissues . The gene-delivery procedure: **A**. A gene of interest (such as YFP or α-MSH) is subcloned into the FIV-derived plasmid, pLionII, and the recombinant pLionII plasmids are used as a part of a three-plasmid system to produce the corresponding viral particles (such as FIV-YFP or FIV-α-MSH). **B**. E11 embryos are illuminated in a dark room and a prominent blood vessel of the CAM, approximately 1 cm below the air sac, is marked with a pencil. An oval window of approximately 5×3 mm is carefully drilled into the eggshell around the marked blood vessel, using a Dremel 300JD multitool with aluminum-oxide grinding stone (Dremel, Polack Supply, Haifa, Israel), and the drilled shell piece is gently removed using a fine forceps. Special care is taken to avoid damaging the underlying eggshell membrane. The egg is then stabilized vertically and recombinant lentiviral particles (2×10^6^ TU in 100 µl PBS) are injected through the transparent eggshell membrane into the blood vessel, using a 30 G needle. **C**. After hot-glue sealing of the eggshell window [54], eggs are returned to the incubator until hatch. Scattered clusters of YFP-expressing cells in the liver are illustrated in green. **D**. The table indicates the hatching rates determined for control non-injected chicks and chicks injected with PBS or viral particles. The hatching rate of injected embryos was 81.8%, which by comparing to the un-injected control with 90% hatching rate, calculated to represent a 9% reduction in hatch. WPRE, woodchuck hepatitis post-transcriptional regulatory element; pCMV, cytomegalovirus promoter.

The procedure used for the introduction of foreign genes into chicken tissues *in ovo* via administration of lentiviral particles is presented in detail in Figure 2. Recombinant lentiviral particles, harboring genes of interest, are injected into a selected CAM vein through the eggshell membrane after removal of a small piece of the calcified shell. The shell is re-sealed and embryos are incubated to hatching. Hatchability rate was reduced by an average of 9% in PBS or viral injected eggs, as compared to non-treated eggs following this procedure (Figure 2D).

### Transgene Expression in Tissues of Hatched Chicks Following *in ovo* Transduction with FIV-YFP

**Figure 3:**
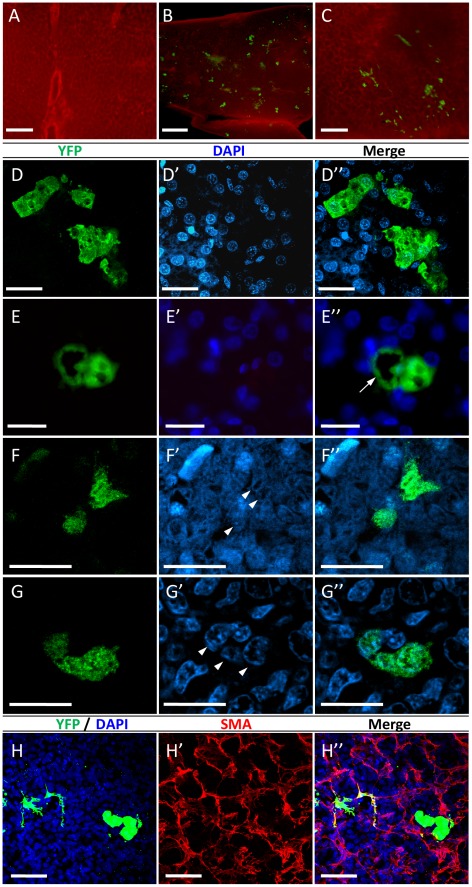
Immunofluorescence analyses of YFP expression in the liver and spleen of post-hatch chicks following *in ovo* FIV administration. Whole-mount immunostaining with anti-GFP antibody (green) and anti-SMA antibody (red) was performed on pieces of livers from: 2-day-old chicks treated with either PBS (**A**) or FIV-YFP (**B**), and from 40-day-old FIV-YFP-treated chicks (**C**). Images were obtained using epi-fluorescent stereomicroscope. Scale bar = 200 µm for A and C, and 0.5 mm for B. Immunostaining of paraffin sections with anti-GFP antibody was performed on liver (**D & E**) and spleen (**F & G**) tissues from 2-day-old chicks treated with FIV-YFP. For each of these sections, DAPI staining (blue) is shown in the corresponding images (**D′–G′**) to indicate cell nuclei. Arrowheads mark nuclei of YFP-expressing cells with splenocyte morphology (F′). Merged YFP and DAPI staining is shown in the corresponding **D′′–G′′**. Images were obtained by confocal microscopy except for **E**, **E′** and **E**′′, which were obtained by epi-fluorescenct microscope. The arrow in **E′′** indicates a YFP-expressing cell with endothelial morphology located next to transduced cells with hepatocyte morphology. **H**. Confocal images of whole-mount immunostained liver sample, using both anti-GFP (**H**) and anti-SMA (**H′**) antibodies, confirmed the association of some of the YFP-expressing cells with blood vessels, which appear in yellow in the merged image (**H′′**). Scale bar = 20 µm (D-G) and 50 µm (H).

Following *in ovo* transduction with FIV-YFP (Figure 2), various tissues from post-hatch chicks were analyzed for reporter gene expression by immunostaining using anti-GFP antibody (which also recognizes YFP that differs from GFP only by a Y66W substitution). As demonstrated in Figure 3, clusters of YFP-expressing cells were detected by whole-mount immunostaining on pieces of liver tissue excised on days 2 and 40 post-hatch (Figure 3B & C, respectively) from FIV-YFP-treated chicks, but not from control chicks treated with PBS (Figure 3A) or with FIV particles, carrying another cDNA (α-MSH, data not shown). Similar clusters of YFP-expressing cells were also detected by immunostaining of paraffin sections of liver from 2-day-old chicks treated with FIV-YFP (Figure 3D & E). Images show YFP staining in cells with characteristic hepatocyte morphology (Figure 3D) as well as cells associated with blood vessels (Figure 3E). Transduction of cells associated with blood vessels in the liver was confirmed by double-staining with anti-GFP and anti-SMA antibodies (Figure 3H). An additional view of YFP-expressing cell clusters, which predominantly have characteristic hepatocyte morphology, is demonstrated in 3D images of liver tissue, stained with anti-GFP (Movie S1) or double stained with anti-GFP and anti-SMA antibodies (Movie S2). Another demonstration of the typical morphology of hepatocytes is provided in Figure S3. These images show that both GFP-positive and negative cells seem similar, as well as hepatic tissue from control non-transduced chicks.

YFP-expressing cells were also observed in spleen sections of FIV-treated chicks (Figure 3F & G). A higher frequency of YFP-expressing cells was observed, but in small clusters of two or three cells each. Examination of paraffin sections from kidney, brain, heart and muscle tissues revealed sporadic YFP expressing cells (data not shown). Yet, the appearance of YFP expression in these tissues was significantly lower compared to liver and spleen.

**Figure 4:**
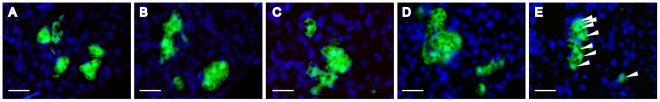
Serial paraffin sections of YFP-positive cell cluster in the liver. A cluster of YFP-expressing cells (green) was identified in five consecutive liver sections (**A–E**) by immunofluorescence with anti-GFP antibody, demonstrating its 3D structure. DAPI staining (blue) indicates cells nuclei. Arrowheads in **E** indicate nuclei of transduced cells. Scale bar = 20 µm.

To estimate the number of cells in a representative YFP positive cluster, several serial sections of paraffin-embedded liver sections were analyzed (Figure 4). The signals were observed at the same position in several serial sections indicating the specificity of the detecting antibody and the three-dimensional structure of the cluster. Given the estimation of 12- µm diameter for a chicken liver cell [25], it can be assumed that most of the nuclei in each of the consecutive 10 µm tissue slices (Figure 4E, arrowheads) represent different cells in the cluster. For a rough estimation of the number of cells in a cluster, the numbers of transduced cells’ nuclei per section in Figure 4A–E were 9, 10, 11, 12, & 8, respectively, resulting in 50 nuclei of YFP-expressing cells.

**Figure 5:**
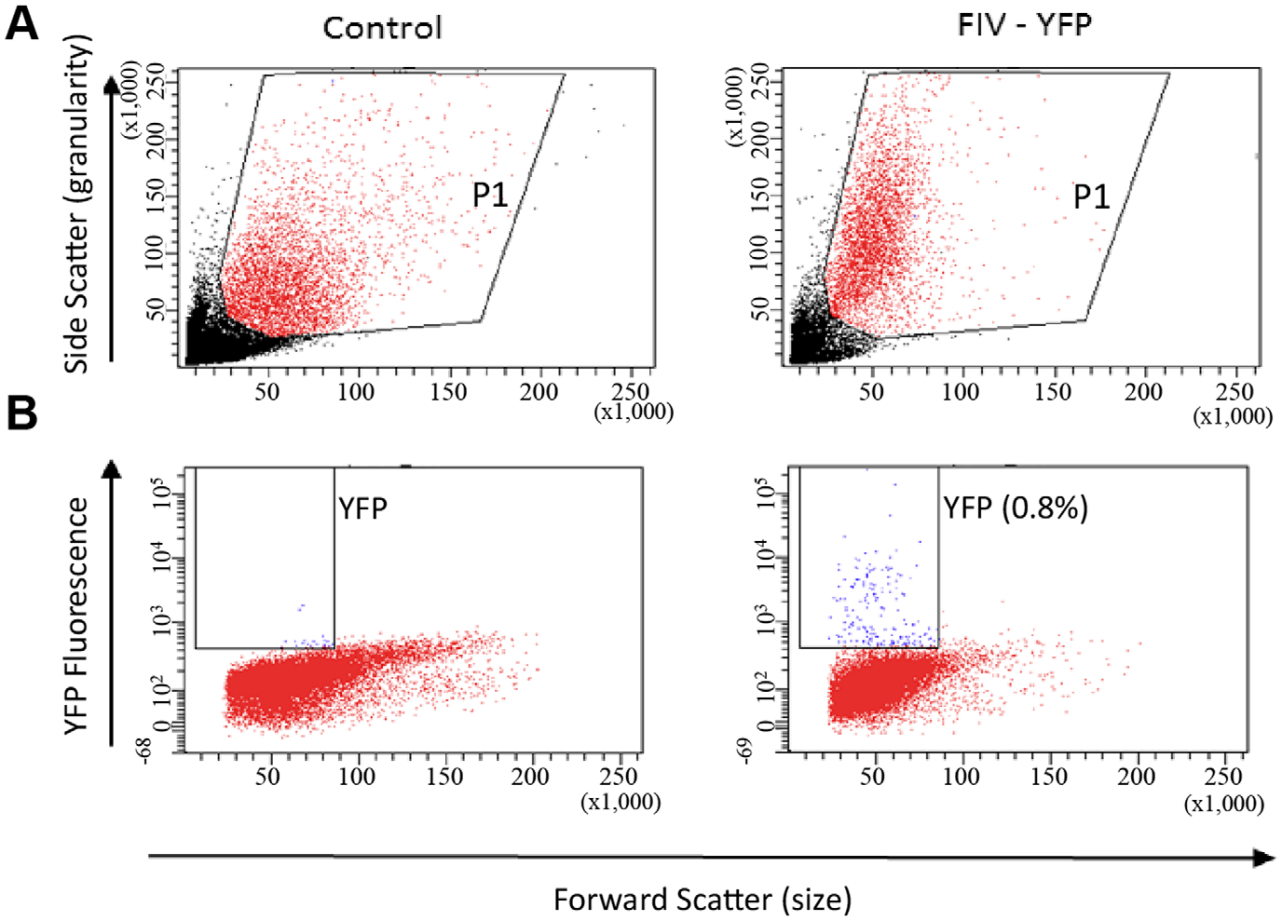
Estimation of transduction efficiency in livers of *in ovo* FIV-YFP transduced chicks by flow cytometry. Liver samples from 2-day-old control chicks treated with PBS (**A**) or FIV-YFP (**B**) were analyzed by flow cytometry. One representative image from the control PBS and FIV-YFP treated chick groups is shown. Cells in each sample were selected (window P1 in A) and YFP fluorescence was analyzed (B). While no obvious signals were obtained in the control samples, the rate of transduced liver cells in the samples from FIV-YFP treated chicks was 0.46±0.19% (n = 3). Blue and red mark the YFP-positive and negative cells, respectively. The sample with the highest transduction rate (0.8%) is presented.

To estimate the proportion of transduced cells in the liver, liver cells were prepared from excised tissue samples and analyzed by flow cytometry. As shown in Figure 5, the average percentage of transduced cells was 0.46±0.19 (n = 3), a proportion similar to that reported in mice following lentiviral transduction *via* tail injection [26].

**Figure 6:**
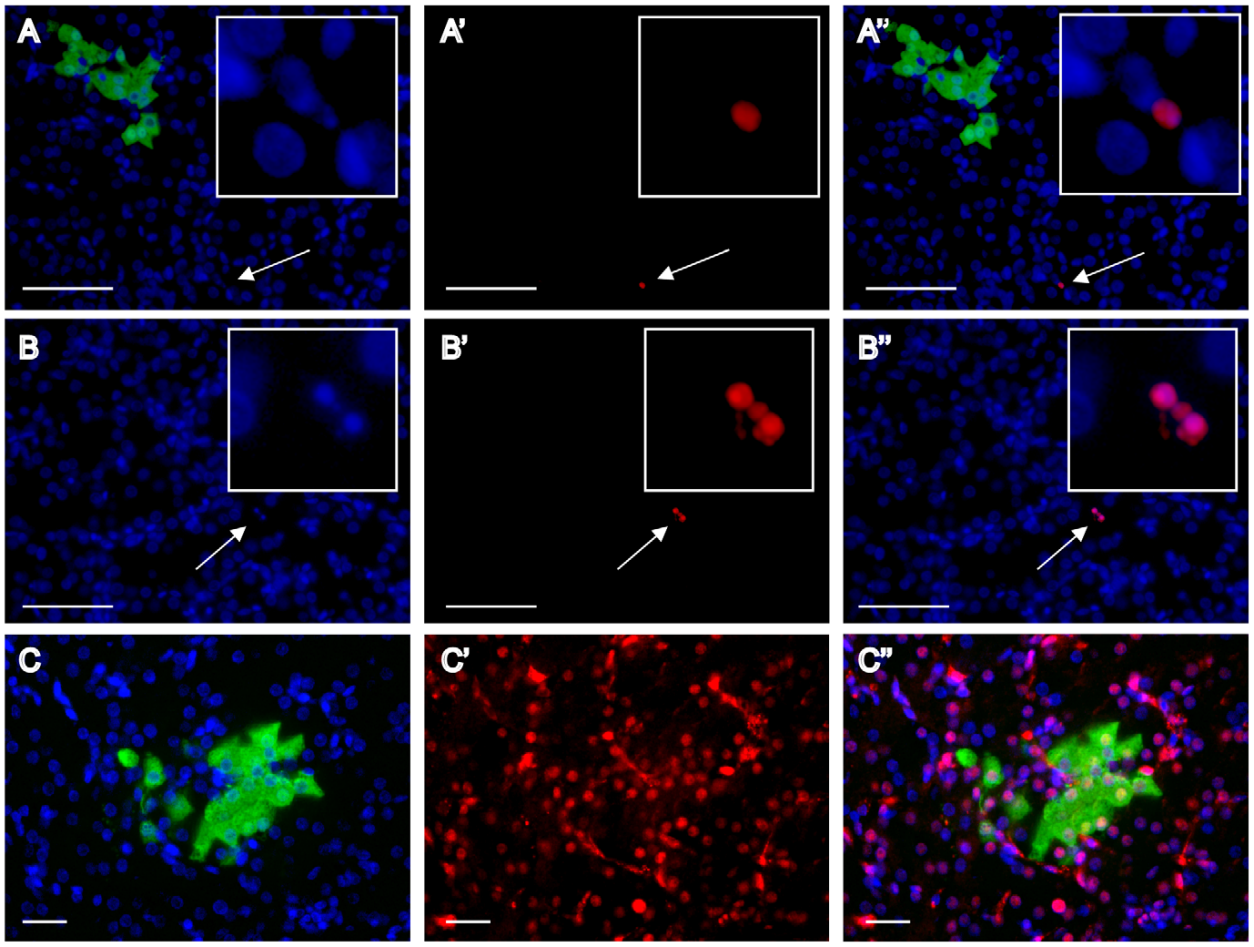
Viability of transduced cells in liver of chicks, as estimated using TUNEL and PCNA. Liver paraffin sections of FIV-treated chicks (**A**) and controls (**B**), immunostained with anti-GFP antibody (green) and DAPI (blue), were analyzed for apoptosis using TUNEL assay (A′, B′, red). Very low incidence of cell death was observed in both groups of chicks. The areas of the TUNEL positive cells were enlarged in the white boxes (inserts). Scale = 100 µm. Liver paraffin sections were immunostained with anti-GFP and DAPI (C, green and blue, respectively) and anti-PCNA (C′, red). Co-labeling of a group of hepatocytes is demonstrated (C′′), indicating actively proliferating FIV-transduced cells. Scale = 50 µm.

Further examinations of liver sections were performed to verify the viability of the transduced cells (Figure 6). The possibility of cell death through apoptosis was examined using TUNEL assay. As demonstrated in Figure 6A & B, only very few TUNEL-positive signals were obtained in sections of either FIV-YFP transduced chicks or controls, respectively. None of these appeared to co-localize with YFP-expressing cells. The images in Figure 6 represent one example of 10 slides that were analyzed.

Immunostaining using anti-PCNA antibody, which is directed against the auxiliary protein of DNA polymerase delta, was performed to indicate cell proliferation. As shown in a representative image in Figure 6C, some PCNA signals were found to co-localize with YFP fluorescence.

The PCNA and TUNEL analyses confirmed viability of the YFP-transduced cells in the hatching chicks. These results are compatible with the detection of YFP signals in pieces of liver excised from 40-day-old chickens (51 days post transduction), analyzed by whole-mount immunostaining (Figure 3C).

Taken together, these data demonstrate that *in ovo* injection of lentiviral particles leads to stable transduction of somatic tissues that can be detected post-hatch, primarily in the liver and spleen.

### Transgene Expression Following Transduction with FIV-α-MSH *in vitro* and *in ovo*

**Figure 7:**
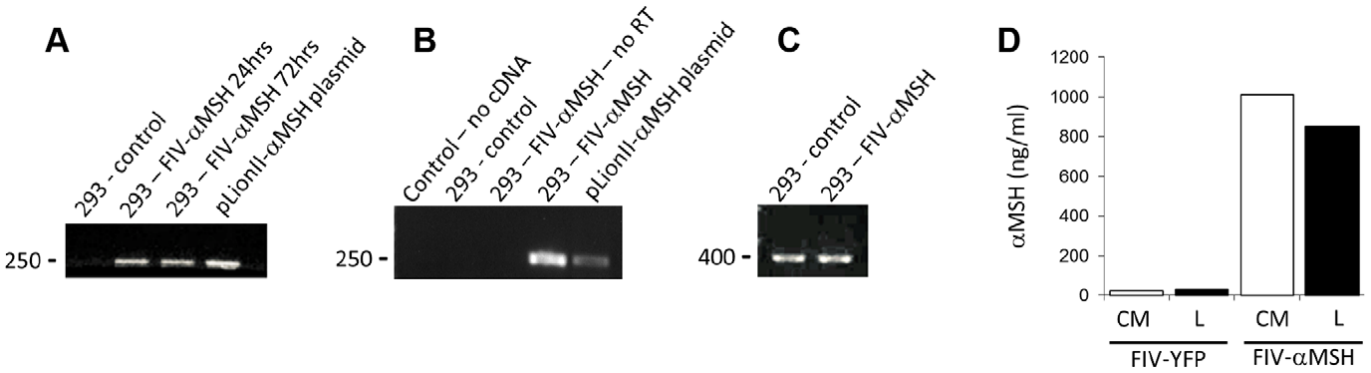
FIV-α-MSH transduction of HEK293T cells: characterization at the DNA, mRNA and protein levels. A . Genomic DNA extracted either before (293T) or 24 and 72 h after transduction of HEK293T cells with FIV-α-MSH (293T-α-MSH) was analyzed by PCR using the recombinant α-MSH-specific primers. The plasmid pLionII- α -MSH was used to demonstrate the expected size fragment. **B**. RNA samples, prepared from similarly treated cell cultures 20 days after transduction, were analyzed by RT-PCR using the same primers. **C**. The integrity of the mRNA in the control and transduced culture cells was demonstrated using the GAPDH primers. **D**. Production and secretion of α-MSH peptide was demonstrated by RIA. α-MSH peptide was detected in cell lysate (L) and culture medium (CM) of HEK293T cells, 10 days after transduction with FIV-α-MSH. No signal was detected in cell lysate or culture medium of control HEK293T cells, transduced with FIV-YFP (293T-YFP).

Once we had successfully observed YFP expression using the CAM-injection approach, we set out to validate our findings by analyzing the expression of a functional gene, encoding the secreted α-MSH peptide. This peptide has pleiotropic effects on energy homeostasis, demonstrated mainly in mammalian species [27]. The presence of α-MSH was first analyzed at the DNA and mRNA levels following transduction of HEK293T cells in culture, with a FIV vector carrying α-MSH (FIV-α-MSH). The α-MSH sequence was detected in genomic DNA samples extracted from cells harvested 24 and 72 h after transduction, using primers specific for the recombinant α-MSH (Figure 7A). A further indication of the specificity of the PCR results was obtained by purification and sequencing of the PCR product (not shown). Expression of α-MSH mRNA in the transduced cells was next examined by RT-PCR. Transcripts of the α-MSH-encoding sequence were detected in transduced HEK293T cell cultures 20 days post-transduction (Figure 7B). The controls included: cDNA with no RT enzyme, a reaction lacking template cDNA, and cDNA from non-transduced cells. GAPDH-specific primers were used as an additional control to demonstrate the integrity of the mRNA samples of the transduced and nontransduced cells (Figure 7C).

Next, production and secretion of a bioactive α-MSH peptide was assessed by RIA (Figure 7D). Cell extracts and conditioned media of HEK293T cells, transduced with FIV-α-MSH, produced RIA-detectable peptide (852 and 1012 ng/ml, respectively). In contrast, no RIA signal could be obtained in control cells transduced with FIV-YFP (Figure 7D). These results indicate our ability to produce active FIV-α-MSH particles, which are capable of directing the production and secretion of α-MSH in transduced cells.

**Figure 8:**
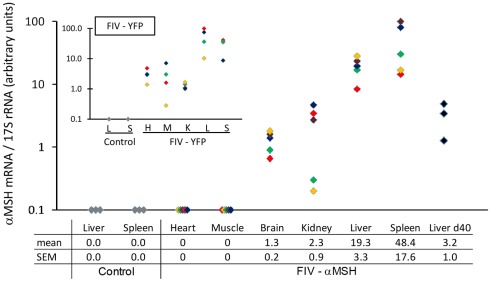
α-MSH expression in tissues of FIV-α-MSH-treated chicks. qPCR analysis of the indicated tissues from 2-day post-hatch chicks was performed following *in-ovo* treatment with FIV-α-MSH particles (2×10^6^ TU/chick), as described in Figure 2. Results of transgene expression in tissues of each chick are indicated by a different color (n = 6). Spleen and liver tissues of chicks treated with FIV-YFP particles served as negative controls. Expression of α-MSH in the liver is also demonstrated in 40-day-old chickens (n = 4). A similar analysis performed using tissues of 2-day-old chicks treated with FIV-YFP, but with YFP-specific primers, is shown in the insert (n = 4). A higher level of transgene expression in the liver and spleen was common to both transgenes. The results are presented as mean ± SEM of triplicate runs (error bars are contained within the symbols).

Following the confirmation of α-MSH expression, production and secretion in cell culture, chick embryos were treated with the same viral particles *in ovo*. Samples from liver, spleen, kidney, brain, heart and muscle were excised two days post hatch and subjected to qPCR using primers that specifically recognize the recombinant α-MSH transcripts and not those of the endogenous POMC (Figure S1). The transgene mRNA was detected in the FIV-α-MSH treated chicks (Figure 8). The level of expression was highest in the spleen and liver (48.4±17.6 and 19.3±3.3, respectively, in arbitrary units). Lower expression was observed in the kidney (2.3±0.9) and brain (1.3±0.2), while no expression could be detected in the heart or muscle. No expression of recombinant α-MSH was detected in tissues collected from control chicks treated with FIV-YFP virus (Figure 8). Moreover, liver samples that were taken from 40 days old chickens (51 days after injection) also demonstrated α-MSH expression, indicating the stability of the transduction. Similar results were obtained also with tissues excised at day 2-post hatch from FIV-YFP treated chicks using YFP-specific primers (Figure 8, insert) with higher relative expression in the liver and spleen.

### Demonstration of Transgene Integration into the Chicken Genome Using CR1-PCR

**Figure 9:**
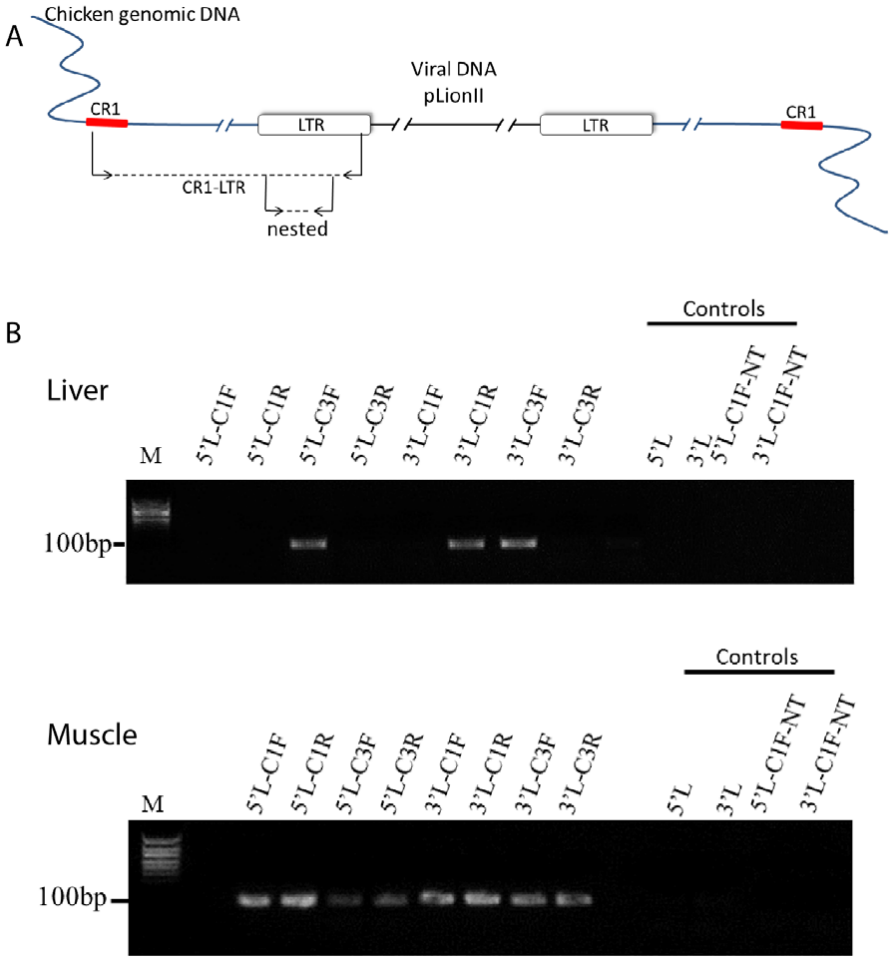
CR1-PCR analysis for genomic integration of YFP in chicken cells, transduced with FIV-YFP vectors. Schematic representation of the two-step PCR approach using the CR1- and LTR-specific primers. Diluted (1∶1000) mixes from the first long PCRs, employing CR1 and LTR primers, were used as templates for short nested PCRs with nested LTR primers. In the absence of genomic integration, no signals are expected in the nested PCR. This is indicated by the control long PCR with no CR1 primers. **B**. Nested PCR products separated on 2% agarose gel, obtained using as templates the first long-PCR DNAs, which were prepared 14 days after *in vitro* transduction with FIV-YFP, from E11 liver and muscle cells. The primers used for the first long PCR are indicated: the LTR primers were 5′LTR (5′L) and 3′LTR (3′L); the CR1 primers were CR1-1F (C1F), CR1-1R (C1R), CR1-3F (C3F) and CR1-3R (C3R). The nested primers are indicated in Materials and Methods. Neither the controls without CR1 primers, nor the control using DNA from non-transduced cell cultures gave a signal. The expected size fragments were 120 bp for the 5′LTR and 110 bp for the 3′LTR. The products were confirmed by sequencing. These results indicate genomic integration of FIV-YFP-derived cDNA in the host chicken cells. M, molecular weight markers.

Primers matching the consensus sequences of the repetitive DNA elements were originally used by Coullin *et al.* [28], for primed *in-situ* labeling analysis. We employed the same primer sequences to detect transgene integration into the chick genome (Figure 9). For preparation of genomic DNA samples, liver and muscle cell cultures of E11 chick embryos were transduced with FIV-YFP, and 14 days later cells were harvested for DNA isolation. As schematically demonstrated in Figure 9A, the amplification of virus-derived LTR sequences in the second step of the PCR critically depended on amplification by the CR1 and LTR primers in the first step. As demonstrated in Figure 9B, PCR product of the expected size was obtained using the nested primers only in genomic DNA samples of the FIV-YFP transduced liver and muscle cell cultures, and not in the non-transduced cell culture controls. Therefore, this analysis demonstrates that transduction of chicken cells results in integration of the FIV-associated transgene into the host cell genome.

Altogether, these data demonstrate the feasibility of our simple and original manipulation in chick embryos for the introduction of foreign genes into chicken somatic tissues, primarily liver and spleen.

## Discussion

We present a novel approach for the introduction of foreign genes into somatic tissues of chickens, by injection of FIV-derived lentiviral particles into the CAM vasculature of embryos. Recent progress has been reported in the successful production of transgenic chicken lines [3]–[6], [9], [29]. However, these approaches are highly complicated and costly [1], [3], preventing their routine laboratory use for studies of gene function. The advantages of the technique described herein stems from its simplicity and high rates of transgene transfer and chick hatchability. The well-documented advantages of lentiviral vectors, particularly their ability to integrate into the host-cell genome and their resistance to downregulation by the endogenous immune system or other cellular mechanisms [30], [31], strongly support the potential of this approach for gene transfer in chickens. This technique provides the first affordable tool for constitutive production of secreted proteins and peptides from the liver, for the study of their long-term effects. This technique is expected to advance molecular-level avian endocrinology research and enable identification of target proteins with importance to agriculture-oriented research, developmental biology and evolution, among other fields. For evolution studies in particular, the chicken provides an important perspective due to its evolutionary position between reptiles and other vertebrates.

Although several types of lentiviruses, similarly pseudotyped with VSV-G, have been previously used in chick embryos [4], the use of FIV-based vectors for the delivery of foreign genes into chicken tissues is demonstrated here for the first time. Therefore, we first characterized the susceptibility of several chick embryonic tissues in culture to FIV transduction. Cells derived from embryonic liver, spleen, kidney, heart, brain and breast muscle tissues were transduced with a low dose of FIV-YFP, and the analysis indicated for the first time the susceptibility of the various chick cell types examined to transduction by FIV-derived particles. Injection of the lentiviral vectors into E11 chick embryos through the CAM vasculature resulted in a more restricted tissue-specific expression profile, with significantly higher relative levels of YFP and α-MSH expression in the liver and spleen, as detected by immunostaining for YFP, and qPCR for both YFP and α-MSH. This transgene-expression profile is in accordance with findings in rodents following lentiviral administration by tail vein injection to adult and neonatal mice [26], [32], [33]. Given the similar tropism for FIV transduction of these cell types *in vitro*, it seems logical to assume that the observed profile of tissue transduction following lentivirus application *in vivo* does not reflect viral tropism, but probably structural differences in the organization of the vasculature of the relevant tissues in the animal. Such a structural explanation could be provided by the fenestrated capillaries characteristic of the spleen, liver and myeloid bone marrow [34], which seem wide enough to facilitate penetration of the lentiviral particles (approximately 100 nm in diameter) [35] from the circulation to the cells of these tissues.

Relatively high efficiency of lentiviral transduction in the liver was demonstrated in a variety of ways: (i) whole-mount immunostaining of pieces of liver tissue from 2- and 40-day-old chickens, (ii) immunostaning of paraffin sections, (iii) 3D confocal imaging, (iv) flow cytometry, and (v) qPCR. This relatively high efficiency of transduction is important, since the liver is among the largest tissues in the body and is highly specialized in processing secreted proteins. Therefore, manipulation of the chicken’s liver by introducing genes encoding secreted proteins will provide a highly useful tool for endocrinological and other studies. Moreover, the pattern of transduction of the liver tissue, characterized by cell clusters, might provide a unique tool to better understand the process of liver regeneration, as a potential model for therapeutically oriented studies of liver diseases [36].

The immunofluorescence analyses, with whole-mount specimens and paraffin sections, indicated transduction of cells with characteristic hepatic morphology as well as with characteristics of other cell types associated with blood vessels. The presence of YFP in cells associated with blood vessels, in addition to hepatocytes, was expected, since viruses were injected into the chick circulation.

While clusters of transduced cells in the liver of 2-day-old chicks were estimated to consist of about 50 cells, clusters in the spleen were much smaller, with only a few cells each. This difference is compatible with the different cell-multiplication rates in these tissues during the period of chick development between E11 and day 2-after hatch [37], and support the hypothesis that the appearance of transduced cells in clusters means that each infected cell transmits the transgene to its daughter cells. The smaller clusters of transduced cells in the spleen were seen at a higher density in each slide (not shown), compatible with the similar overall range of expression level obtained in the qPCR of the spleen and liver with the YFP and α-MSH specific primers. Furthermore, flow cytometry analysis demonstrated 0.46±0.19% infected cells in the liver, similar to the frequencies reported following injection of lentiviruses into mice tails [26].

The pACTH1-17 expression cassette used in the present study to generate the pLionII- α-MSH construct and the FIV-α-MSH lentiviral particles has been used previously to direct the production and secretion of a bioactive α-MSH peptide in rodents [23], [38], [39]. The production of a bioactive peptide was demonstrated in those reports through α-MSH protective effect against liver fibrosis [39], ocular autoimmune diseases [38], as well as experimental encephalomyelitis [23]. Here we demonstrate that FIV-α-MSH particles can transduce HEK293T cells, leading to the detection of exogenous α-MSH sequences by PCR, RT-PCR, and qPCR. In addition, we provide the first RIA identification of an active α-MSH product of the FIV-α-MSH. RIA detection of α-MSH peptides in both cell lysate and conditioned medium of HEK293T cells, transduced with FIV-α-MSH, demonstrate that α-MSH production and secretion can be directed through FIV transduction. In mammals, α-MSH has been shown to participate in the control of energy homeostasis, as it is a primary target of the satiety hormone leptin [27], and to be involved in pigmentation and regulation of the immune response [40], [41]. In chickens, data on the physiological roles of α-MSH were obtained in our and others’ laboratories, mainly by characterization of short-term physiological effects following a single administration of α-MSH or its synthetic analogs to young chicks [42]–[48]. Therefore, we strongly believe that the technique presented here will dramatically advance the study of α-MSH in chickens, as a proof of concept, by providing an assay system for long-term studies *in vivo*.

Integration of FIV-derived cDNA into the host cell genome was demonstrated using a CR1-PCR approach, which makes use of repetitive genomic sequences to amplify the ends of the viral LTR sequences. This approach is based on the Alu-PCR protocols used in our previous study to demonstrate genomic integration of FIV-derived sequences in mammals [16], and is applied here to the chicken genome for the first time, by making use of previously characterized CR1 consensus sequences [28]. The CR1 repeats are the most abundant repeat family in avian species, belonging to long interspersed nuclear elements with more than 200,000 copies, accounting for about 80% of the chicken interspersed repeats [49]. They are significantly less abundant than the Alu sequences in the human genome, estimated to be about a million per haploid genome [50]. The fact that this approach gave the expected results despite this differential abundance might support the speculation that the spread of CR1 sequences, and possibly also the integration of viral vectors, has some bias in favor of sites of transcriptionally active chromatin. Notably, demonstration of transgene integration into the chick genome supports the stability and robustness of our *in ovo* transduction approach, and is in accordance with our findings of sustained expression of YFP and α-MSH in 40-day-old chickens.

Future use of lentiviral particles to deliver transgenes using our approach might entail using liver-specific promoters, such as the human α-antitrypsin (hAAT) [51], to restrict transgene expression to the liver. The advantages of using these promoters are twofold: first, it reduces possible undesired effects due to expression in other tissues, and second, their nonviral origin reduces the chances of downregulation of the transgene [32], which was reflected to some extent in the current study by the lower qPCR signals obtained for the livers of 40-day-old vs. 2-day-old chickens. We have previously demonstrated prolonged and stable hAAT-driven gene expression in liver cell lines and murine livers using FIV vectors [16], [33]. In addition, we have reported efficient hAAT-driven gene expression in chicken liver using a naked DNA-delivery system [52]: by using the hydrodynamics-based gene-transfer method to direct gene expression in chick liver, we detected physiologically significant levels of transgene (human coagulation factor IX) in the circulation using the hAAT promoter [52]. This naked DNA-transfer technique does not involve DNA integration into the host-cell genome, and the introduced plasmid persisted in its original episomal form [53]. Nevertheless, that report indicated that the hAAT promoter can be used to drive expression of foreign genes in chickens.

In summary, we have established a new approach for the transfer of genes to chickens. The use of lentiviral vectors enables integration of transgenes into the host-cell genome, while injection into the CAM vasculature provides unique accessibility to the chick embryo. The technique is best suited for the study of endocrinology in avian species, by directing genes encoding secreted hormones and peptides to the liver tissue. This procedure should also be useful for experiments in developmental biology, enabling the monitoring of small subpopulations of transgenic cells within a tissue. In addition, it should be possible to co-transduce a gene of interest and a reporter gene and study the effects of this manipulation in a background of non-transduced cells, on either cell morphology as seen under a microscope, or on gene expression as detected by double-immunostaining. Given the great difficulty involved in gene delivery to birds, the ability to introduce genes coding for secreted proteins into the liver is expected to be highly useful and provide new opportunities for agricultural and academic research in these animals. Being highly specialized in processing secreted proteins and among the largest organs of the body, the liver is an optimal target for producing and secreting exogenous proteins of interest for the study of gene function in chickens.

## Supporting Information

Figure S1 **Position of primers used for α-MSH amplification.** The positions of the qPCR and RT-PCR primers are shown, relative to the sequence of the recombinant α-MSH gene. Note that for both primer sets, the forward primers match the pLionII backbone sequence, thus rendering specificity of the PCR amplification for recombinant α-MSH only.
(TIF)

Figure S2 **Flow cytometry of primary cultures from E11 chick embryos following** ***in vitro*** **transduction with FIV-YFP particles.** The indicated primary cultures of E11 chick embryos were transduced with FIV-YFP at a MOI of 0.1, as described in the Materials and Methods and Figure 1. The rate of transduction was analyzed by flow cytometry in comparison to control non-transduced cells. YFP-positive cells are indicated in blue. The rest of the cells are indicated in red.
(TIF)

Figure S3 **Immunofluorescence analysis of YFP expression in the liver of post-hatch chicks, following** ***in ovo*** **administration of vehicle or viral particles encoding YFP.** Paraffin sections of chicks liver tissues were subjected to immunofluorescence, using anti-GFP antibody (green). Similar tissue and cell morphology was observed in both YFP-expressing cells and non-transduced cells (A), as well as in liver cells of vehicle treated chicks (B). Scale bar = 100 µm
(TIF)

Movie S1 **3D confocal illustration of YFP-positive hepatocyets: whole-mount staining with anti-GFP antibody.** Nuclei were stained with DAPI.
(AVI)

Movie S2 **3D confocal illustration of YFP-positive cells in the liver, demonstrating their association with blood vessels: whole-mount staining with anti-GFP and anti-SMA antibodies.** Nuclei were stained with DAPI.
(AVI)
