# Correction: Gene Transfer to Chicks Using Lentiviral Vectors Administered via the Embryonic Chorioallantoic Membrane

**DOI:** 10.1371/annotation/1cdd9e97-8652-415e-a79b-1caa5298c684

**Published:** 2012-07-10

**Authors:** Gideon Hen, Sara Yosefi, Dmitry Shinder, Adi Or, Sivan Mygdal, Reba Condiotti, Eithan Galun, Amir Bor, Dalit Sela-Donenfeld, Miriam Friedman-Einat

There were errors in the legend of Figure 5, "Estimation of transduction efficiency in livers of in ovo FIV-YFP transduced chicks by flow cytometry." The complete, correct Figure 5 is: "Liver samples from 2-day-old control chicks treated with PBS (control) or lentiviruses (FIV-YFP) were analyzed by flow cytometry. One representative image from the control PBS and FIV-YFP treated chick groups is shown. Cells in each sample were selected (window P1 in A) and YFP fluorescence was analyzed (B). While no obvious signals were obtained in the control samples, the rate of transduced liver cells in the samples from FIV-YFP treated chicks was 0.46±0.19% (n = 3). Blue and red mark the YFP-positive and negative cells, respectively. The sample with the highest transduction rate (0.8%) is presented." 

