# Correction: Gene Transfer to Chicks Using Lentiviral Vectors Administered via the Embryonic Chorioallantoic Membrane

**DOI:** 10.1371/journal.pone.0138629

**Published:** 2015-09-14

**Authors:** Gideon Hen, Sara Yosefi, Dmitry Shinder, Adi Or, Sivan Mygdal, Reba Condiotti, Eithan Galun, Amir Bor, Dalit Sela-Donenfeld, Miriam Friedman-Einat

There is an affiliation missing for the fourth author. Adi Or is also affiliated with #2 Koret School of Veterinary Medicine, The Robert H. Smith Faculty of Agriculture, Food & Environment, The Hebrew University of Jerusalem, Rehovot, Israel
