# Supplementary figures and images for: Gene Transfer to Chicks Using Lentiviral Vectors Administered via the Embryonic Chorioallantoic Membrane
Source: PLoS One. 2012 May 11;7(5):e36531. doi: 10.1371/journal.pone.0036531 (PMC3350527; doi:10.1371/journal.pone.0036531)

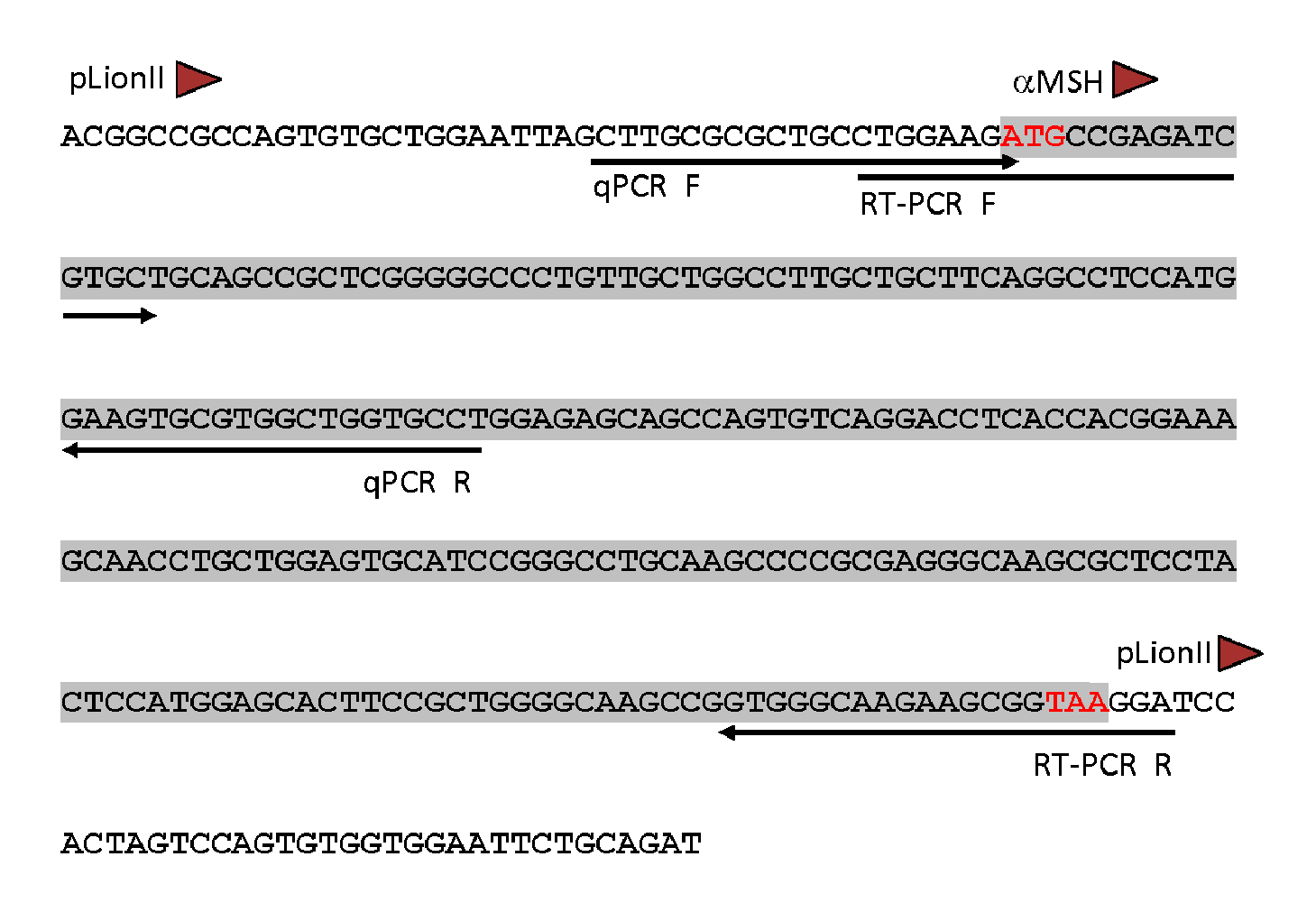

Supplement: Figure S1 — Position of primers used for α-MSH amplification. The positions of the qPCR and RT-PCR primers are shown, relative to the sequence of the recombinant α-MSH gene. Note that for both primer sets, the forward primers match the pLionII backbone sequence, thus rendering specificity of the PCR amplification for recombinant α-MSH only. (TIF) [file pone.0036531.s001.tif]

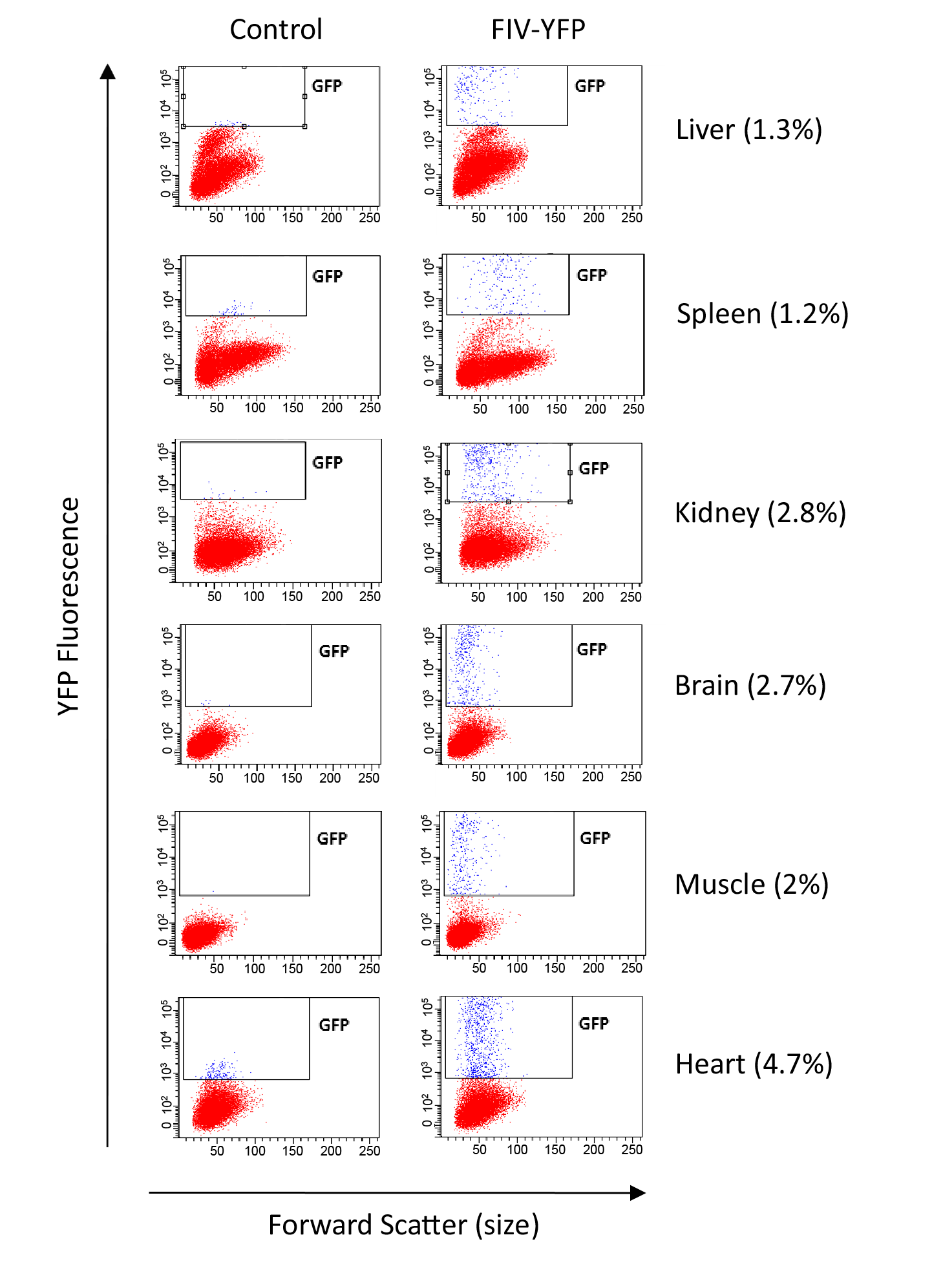

Supplement: Figure S2 — Flow cytometry of primary cultures from E11 chick embryos following in vitro transduction with FIV-YFP particles. The indicated primary cultures of E11 chick embryos were transduced with FIV-YFP at a MOI of 0.1, as described in the Materials and Methods and Figure 1. The rate of transduction was analyzed by flow cytometry in comparison to control non-transduced cells. YFP-positive cells are indicated in blue. The rest of the cells are indicated in red. (TIF) [file pone.0036531.s002.tif]

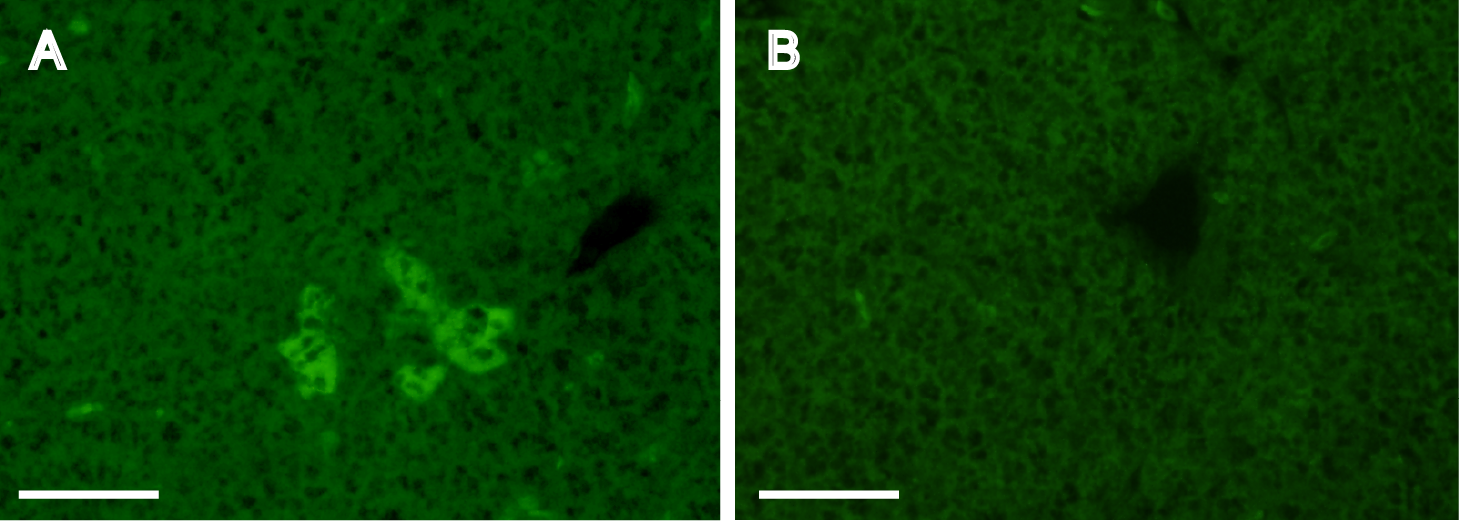

Supplement: Figure S3 — Immunofluorescence analysis of YFP expression in the liver of post-hatch chicks, following in ovo administration of vehicle or viral particles encoding YFP. Paraffin sections of chicks liver tissues were subjected to immunofluorescence, using anti-GFP antibody (green). Similar tissue and cell morphology was observed in both YFP-expressing cells and non-transduced cells (A), as well as in liver cells of vehicle treated chicks (B). Scale bar = 100 µm (TIF) [file pone.0036531.s003.tif]
